# Spatiotemporal atmospheric *in-situ* carbon dioxide data over the Indian sites-data perspective

**DOI:** 10.1038/s41597-024-03243-x

**Published:** 2024-04-16

**Authors:** Mahesh Pathakoti, Mahalakshmi D.V., Sreenivas G., Arun Shamrao Suryavanshi, Alok Taori, Yogesh Kant, Raja P., Rajashree Vinod Bothale, Prakash Chauhan, Rajan K.S., P. R. Sinha, Naveen Chandra, Vinay Kumar Dadhwal

**Affiliations:** 1 National Remote Sensing Centre, Indian Space Research Organisation (ISRO), Department of Space, Hyderabad, 500037 India; 2 Lab for Spatial Informatics (LSI), International Institute of Information Technology (IIIT), Hyderabad, 500032 India; 3 Department of Physics, Jawaharlal Nehru Technological University Hyderabad (JNTU-H), Hyderabad, 500085 India; 4 Regional Remote Sensing Centre, NRSC, ISRO, Nagpur, 440033 India; 5 Indian Institute of Remote Sensing (IIRS), ISRO, Department of Space, Dehradun, 248001 India; 6 Indian Institute of Soil and Water Conservation (IISWC), Research Centre (RC), Ooty, The Nilgiris 643001 India; 7 Indian Institute of Space Science and Technology (IIST), Valiamala, 695547 India; 8 Research Institute for Global Change (RIGC), Japan Agency for Marine-Earth Science and Technology (JAMSTEC), Yokohama, 2360001 Japan; 9 National Institute of Advanced Studies (NIAS), Indian Institute of Science (IISc) campus, Bengaluru, 560012 India; 10 Present Address: Indian Institute of Tropical Meteorology, Pune, 411008 India; 11 Present Address: ICAR-IISWC, RC, Odisha, Koraput 763002 India

**Keywords:** Atmospheric chemistry, Environmental impact

## Abstract

In the current study, atmospheric carbon dioxide (CO_2_) data covering multiple locations in the Indian subcontinent are reported. This data was collected using a dedicated ground-based *in-situ* network established as part of the Geosphere-Biosphere Programme (CAP-IGBP) of the Climate and Atmospheric Processes of the Indian Space Research Organisation (ISRO). Data are collected over Ponmudi, Ooty, Sriharikota, Gadanki, Shadnagar, Nagpur, and Dehradun during 2014-2015, 2017–2020, 2012, 2011–2015, 2014–2017, 2017 and 2008–2011, respectively. The atmospheric CO_2_ generated as part of the CAP−IGBP network would enhance the understanding of CO_2_ variability in different time scales ranging from diurnal, seasonal, and annual over the Indian region. Data available under this network may be interesting to other research communities for modeling studies and spatiotemporal variability of atmospheric CO_2_ across the study locations. The work also evaluated the CO_2_ observations against the Model for Interdisciplinary Research on Climate version 4 atmospheric chemistry-transport model (MIROC4-ACTM) concentrations.

## Background & Summary

Carbon dioxide (CO_2_) emissions from human activity are one of the leading causes for the complicated issue known as “human-induced climate change”. Other activities that release greenhouse gases (GHGs) into the atmosphere include burning fossil fuels^1^. CO_2_ contributes about 64% of the total radiative forcing created by other long-lived GHGs^2^. The accelerating CO_2_ mixing ratios were attributed to the land use land cover (LU/LC) changes, biological and human-induced process. The amount of CO_2_ released into the atmosphere by human activity and the rate at which concentrations increase estimate the global carbon budget^3,4^. Burning fossil fuel and LU/LC changes have increased CO_2_ by 40%^5,6^. This gas has been consistently increasing since pre-industrial times and crossed 400 ppm of daily mean in 2013 at the global reference site of Mauna Loa, Hawaii^7^. During 2013, in India, CO_2_ emission was found to be 0.96 Ton/capita (http://www.iaea.org/inis/aws/eedrb/data/IN-enemc.html). An increase in atmospheric CO_2_ from industrial or human activity is the most significant contributor to possible anthropogenically induced global climate change^8^. Local meteorological conditions such as air temperature and moisture affect the diurnal and seasonal cycle^9^. The variability of environmental factors may significantly affect regional and global climate^10^, especially the radiative forcing, via the terrestrial carbon cycle’s biogeochemical pathways. Since CO_2_ mixing ratios in the atmosphere are strongly affected by photosynthesis, respiration, biomass, fossil fuel burning, and the air-sea exchange process, *in-situ* atmospheric CO_2_ measurements are essential data for understanding the carbon cycle^11^.

High precision *in-situ* measurements are more reliable concerning the better representation of GHG concentrations over a region^12^. An amalgamation of long-term observations from *in-situ*, remote sensing, and model-simulated atmospheric CO_2_ concentrations would significantly contribute toward understanding the climate system. Development of measuring infrastructure has advanced to perform high precision measurements of GHGs while meeting the World Meteorological Organisation (WMO) standards^13^. To understand the CO_2_ variability and the underlying dynamics over different parts of India, several researchers such as the National Institute of Oceanography (NIO), Indian Institute of Tropical Meteorology (IITM, Pune) and Physical Research Laboratory (PRL, Ahmedabad)^14,15^ are measuring high-precision CO_2_ measurements. The ground based atmospheric CO_2_ concentrations network over Indian region established by various research centers^16,17^. Huo *et al*.^18^ reported fossil fuel and cement industry emissions at the city level covering 1500 cities in 46 countries.

The current study presents first data on atmospheric CO_2_ concentrations recorded from different locations in India through a well-established CO_2_ network by the Climate and Atmospheric Processes of the Indian Space Research Organisation (ISRO)’s Geosphere−Biosphere Programme (CAP-IGBP). The National Remote Sensing Centre (NRSC), ISRO, built this network to resolve space−time diurnal and seasonal variability and construct a prospective record of atmospheric CO_2_ in the country. By utilising this *in-situ* CO_2_ data from the CAP-IGBP network, an integrated study with the remote sensing and model simulated atmospheric CO_2_ concentrations, Mahesh *et al*.^19^ carried out a study to assess the diurnal and seasonal variability over the Indian sites as a function of different geographical locations. The CO_2_ sensor installation covers various geographical features, including coastal, high-altitude, and dry climate conditions. The data is necessary to comprehend the CO_2_ variations spatially and temporally across India. Data available under this network may be interesting to modeling research communities that aim to adjust the uncertainties resulting from the model simulation. For climate projections to reflect pertinent temporal scales more accurately, the models must be validated and refined based on global GHG measurements. Thus, the *in-situ* measurements are decisive for understanding the carbon cycle and validating the satellite retrievals.

The present paper aims to report the atmospheric CO_2_ *in-situ* data collected over the different geographical locations of the Indian stations. This paper describes the features of atmospheric CO_2_ monitoring stations, common data collecting protocols, procedures employed to generate dry atmospheric CO_2_, and standard calibration methods. Data has potential in resolving the diurnal and seasonal variability as a function of geographical location. The influence of meteorological parameters, especially winds and precipitation have significant impact on the distribution of CO_2_ concentration^9^. The high-altitude stations namely Ooty and Ponmudi are two contrasting sites controlled by the boundary layer processes, which can be studied in detail. The CO_2_ concentration changes among different sites, therefore studies can be carried out by considering factors such as monsoons, altitude, anthropogenic emissions, and land cover type. These datasets are collected with consistent inter-sensor calibration and using the National Oceanic Atmospheric Administration (NOAA) calibration cylinders (CC). The high-quality CO_2_ observations are on high demand especially from fast growing economy India for accurately understanding sources/sinks, their magnitude and spatiotemporal variability using atmospheric inversion. Such estimation will be helpful to develop effective strategies to mitigate CO_2_ emissions.

## Methods

### Overview

**Fig. 1:**
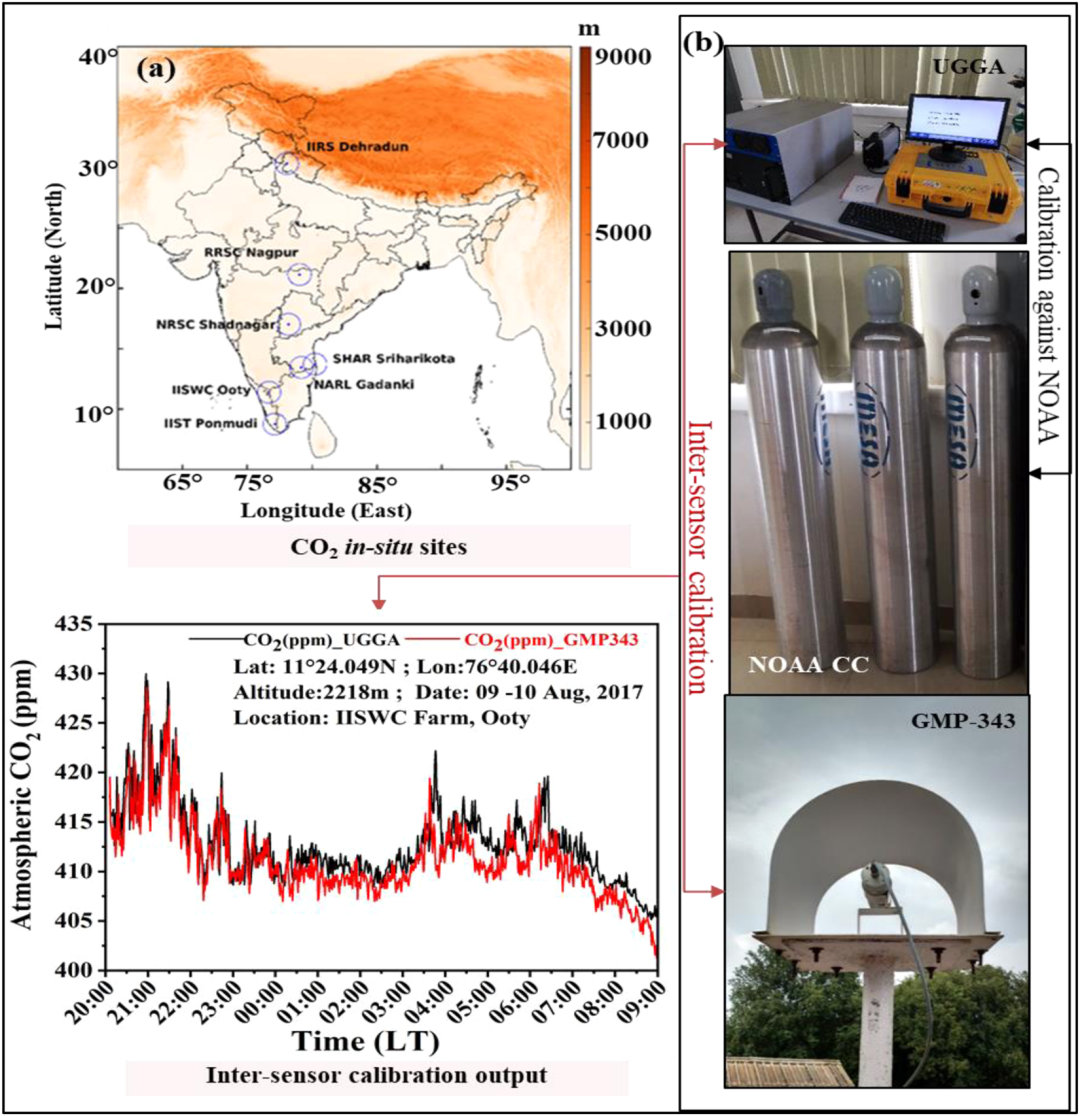
Workflow illustration of atmospheric CO_2_ datasets creation **a**) Study site overlaid on the Digital Elevation Model (DEM) **b**) stages of calibration.

A Vaisala GMP-343 CO_2_ sensor probe through Campbell data loggers was used to collect continuous ambient CO_2_ observations from seven Indian locations, as depicted in Fig. 1. GMP-343 instruments, which works on non-dispersive infrared (NDIR) technology^20^ are set up at the observation location at various time scales. Consequently, the data were intercalibrated using standard calibrated greenhouse gas analyser (GGA) equipment, with biases included.

**Fig. 2:**
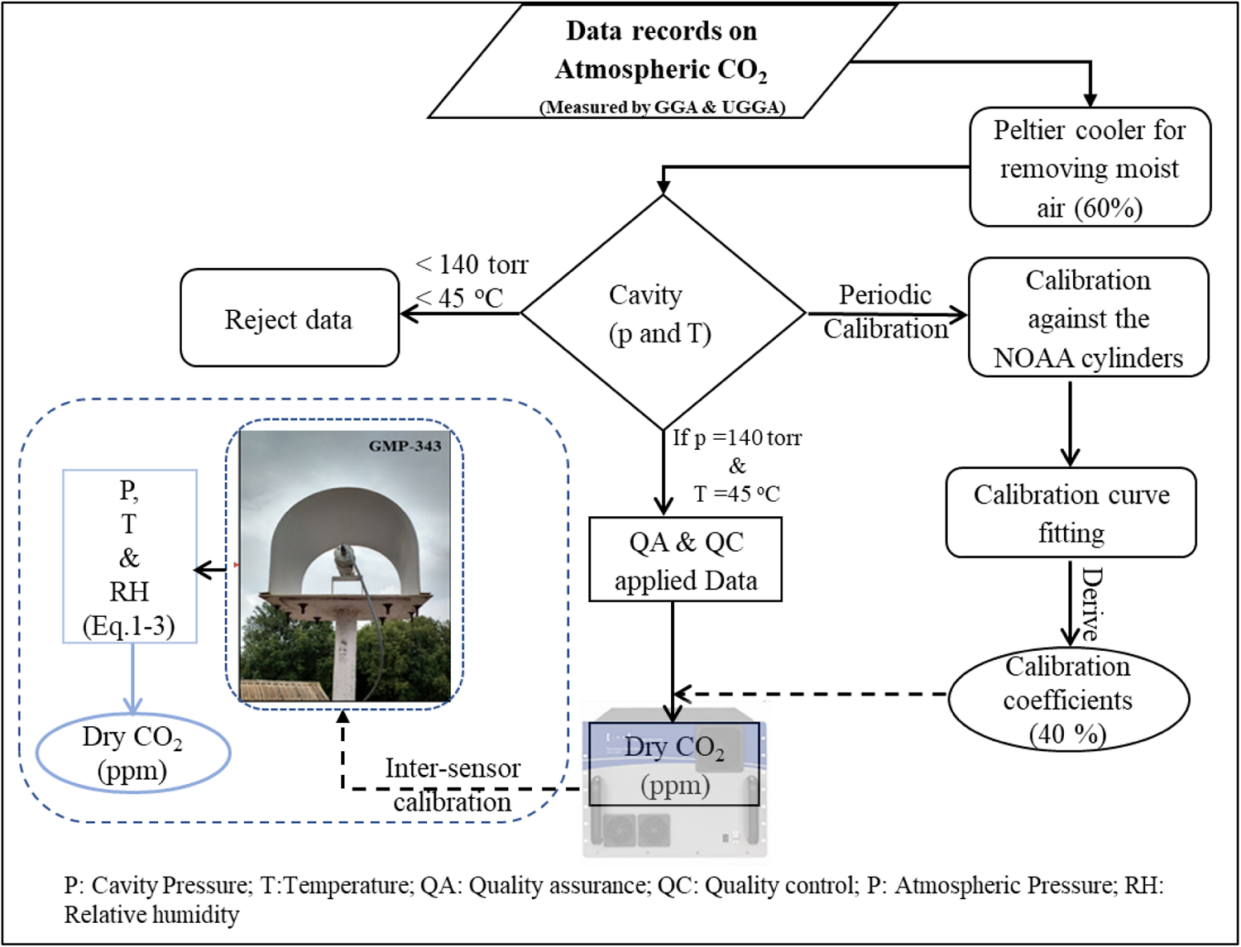
Flow chart of data collection layout from the GGA, UGGA and GMP-343 CO_2_ sensors at the observational sites.

The bias correction was applied linearly from the installation to the calibration date. At each measuring station, the atmospheric CO_2_ observations were collected with 5-minute temporal resolution and integrated to 60-minute. Using the GGA continuous CO_2_ observations were collected from Shadnagar at a temporal frequency of 1 Hz from 2014 to 2017. The Ultraportable GGA (UGGA) is a sophisticated device that simultaneously measures CO_2_, CH_4_, and H_2_O and is also purchased from Los Gatos Research Inc. It also uses a performance-improving off-axis spectroscopy method. True wavelength scanning is used by the enhanced off-axis integrated cavity output spectroscopy (OA-ICOS) technique to capture completely resolved absorption line shapes. A longer effective path length than a typical along-axis setup is made possible by the laser’s off-axis alignment on the highly reflective mirrors inside the instrument chamber. This allows for the extraction of absorption line shapes with higher resolution. To investigate the effects of pressure, drop within the cavity, possibly caused by choked filters, on the absorption line spectra, the raw data were evaluated for cavity pressure and temperature variations. Using measurements of H_2_O, the analyzer adjusts CO_2_ and CH_4_ values for dry air conditions. By removing up to 60% of the ambient H_2_O through a Peltier cooler setup before the air is allowed to enter the GGA, the relatively high concentration of H_2_O in ambient air, which may cause a significant error in such corrections, is reduced. The measuring setup of GGA is given in Mahesh *et al*.^12^ and the data collection layout are described in Fig. 2. Studies show reliable results using these sensors in atmospheric studies^21,22^.

### Calibration

As shown in Table 1, the 3-span calibration gases from the NOAA are utilized for the periodic calibration of GGA and UGGA analyzers. These analyzers are well calibrated against the NOAA CO_2_ spans to evaluate the instrument’s precision and accuracy. The NOAA CO_2_ cylinders are highly accurate while meeting the WMO standards with a reproducibility of ±0.02 ppm. Reproducibility is defined as the consistency of measurements by different time periods using the same measuring equipment. The accuracy in the data file represents the degree of uncertainty which is used for assessment of the quality of the records. The precision and accuracy of CO_2_ were, respectively, 0.078 ppm and 0.101 ppm for sample averaging time of 10 seconds. As shown in Fig. 1, except at the Shadnagar location, all other measuring locations are installed with the Vaisala GMP343 instruments, which were well calibrated against the precision UGGA equipment (make: ABB-Los Gatos Research, U.S.A) and subsequently adjusted the bias in the GMP-343 measured atmospheric CO_2_ data. No additional temperature or pressure adjustments are needed for stations close to mean sea level^23^. Atmospheric CO_2_ concentrations are measured with a portable UGGA of CH_4_/CO_2_/H_2_O analyzer at Ooty station is used for the inter-sensor calibration. UGGA works on off-axis integrated cavity output spectroscopy (ICOS) to measure atmospheric CO_2_ concentrations with laser absorption technology. The precision of this analyzer for CO_2_ measurements are <0.30 ppm^24–26^. As the GMP-343 instruments does not account for ambient moisture, hence the present study implemented the standards empirical equations to remove the water vapour influence and reported in the dry atmospheric CO_2_ concentrations. Detailed air sampling system, calibration and inter-sensor comparison strategy is given in Mahesh *et al*.^12^.

**Table 1:** NOAA calibration span gases.

S.No	Cylinder ID	CO_2_NOAA_.(ppm)	Reproducibility (ppm)
1	CB09852	353.17	±0.02
2	CC718409	404.53	±0.02
	CC718425	448.44	±0.40
**Specifications of UGGA Precision and Accuracy**			
**Precision**		**Accuracy**	**Reference**
78 ppb		101 ppb	NOAA Calibration Cylinder

**Fig. 3:**
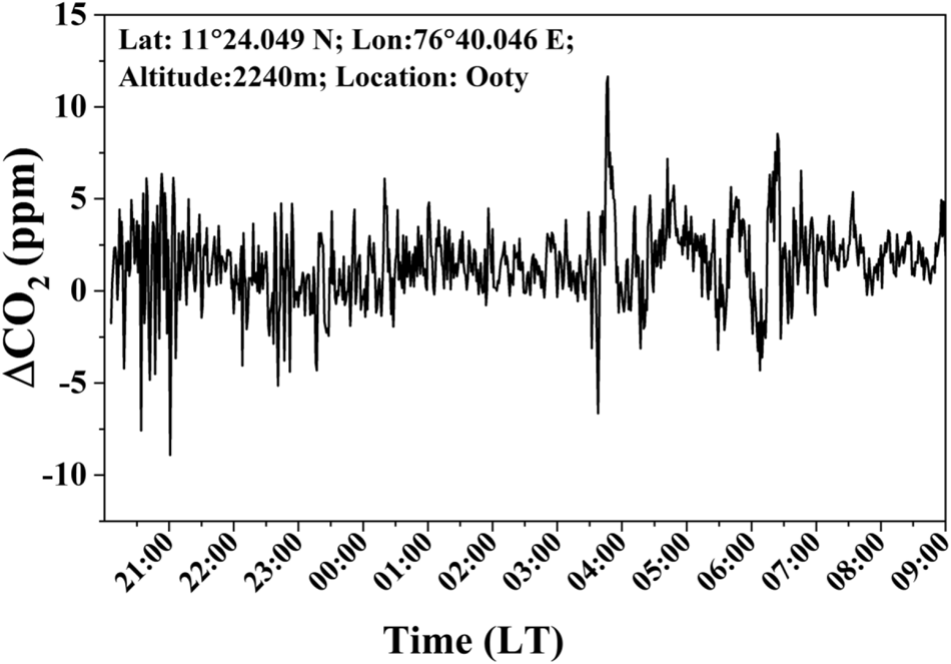
A 12-hour inter-sensor calibration of GMP-343 CO_2_ sensor against high precision UGGA sensor during 9-10 August 2017.

Table 2 provides inter-sensor calibration for every location. As shown in Fig. 3, the GMP-343 sensor functions accurately with an accuracy of 0.62%, as evidenced by the strong correlation between UGGA and GMP-343, which has a root mean square error of 2.57 ppm^19^.

**Table 2:** Calibration of GMP-343 CO_2_ sensors at study locations against UGGA instrument.

Station Name	Date of calibration	Reference data	RMSD (ppm)	Mean (ppm)	Accuracy (%)
NARL, Gadanki	$\frac{1{7}^{{\rm{th}}}}{1{8}^{{\rm{th}}}}$ March 2015	High precision UGGA	$\frac{23.19}{24.53}$	374.56	6.0
RRSC, Nagpur	$\frac{1{7}^{{\rm{th}}}}{1{8}^{{\rm{th}}}}$ March 2015		$\frac{8.30}{14.73}$	389.22	2.0
IISWC, Ooty	09-10^th^ August 2017		2.57	411.25	0.60
IIST, Ponmudi	$\frac{1{7}^{{\rm{th}}}}{1{8}^{{\rm{th}}}}$ March 2015		$\frac{24.54}{25.06}$	372.79	6.50
SHAR, Sriharikota	15^th^ January 2014	Against calibration reference 370 ppm	3.2	373.20	1.0
NRSC Shadnagar	10^th^ March 2015	NOAA CO_2_ references	0.11 ppm	402.92	<0.25

### Atmospheric CO_2_ water vapour correction

Since the GMP-343 operates on NDIR technology, initially the measured atmospheric CO_2_ records are corrected with the ambient temperature and pressure for the high-altitude stations using ideal gas equation as described in^21^. In the data files, the GMP-343 instrument reports atmospheric CO_2_ concentrations without accounting the water vapour. Therefore, using the Wagner and Pruss^27^ equations, the GMP-343 measured wet atmospheric CO_2_ concentrations were corrected to dry atmospheric CO_2_ concentrations. The following are the empirical formulas for calculating the ratio of atmospheric CO_2_ concentration in wet and dry conditions. Except for the Shadnagar site, all other measurement location’s dry air CO_2_ concentrations are estimated using Wagner and Pruss^27^ Eq. (1-3). However, in the Shadnagar site, the high-precision greenhouse gas analyzer will remove 60% of water vapor through its Peltier cooling system. To remove the other 40% water vapour influence, a three-point standard calibration curve is established between measured CO_2_ against the known CO_2_ concentrations using the WMO certified NOAA supplied calibration spans as summarized in Table 1. Further, zero calibration is also applied to adjust the instrument bias. A detailed dry correction method is also discussed in the previous studies by Mahesh *et al*.^19^ and Sharma *et al*.^21,23^.

1 $${\rm{ln}}\left(\frac{{\rm{p}}}{{{\rm{p}}}_{{\rm{c}}}}\right)=\frac{\left({{\rm{a}}}_{1}{\rm{\tau }}+{{\rm{a}}}_{2}{{\rm{\tau }}}^{1.5}+{{\rm{a}}}_{3}{{\rm{\tau }}}^{3}+{{\rm{a}}}_{4}{{\rm{\tau }}}^{3.5}+{{\rm{a}}}_{5}{{\rm{\tau }}}^{4}+{{\rm{a}}}_{6}{{\rm{\tau }}}^{7.5}\right){{\rm{T}}}_{{\rm{c}}}}{{\rm{T}}}$$

2 $${\rm{Relative}}\,{\rm{Humidity}}\,\left({\rm{RH}}\right)=\left(\frac{{\rm{e}}}{{{\rm{e}}}_{{\rm{s}}}}\right)\times 100$$

3 $${\rm{Dry}}\,\left({{\rm{CO}}}_{2}\right)=\frac{{\rm{wet}}\,\left({{\rm{CO}}}_{2}\right)}{1-\left(0.01\times {\rm{e}}\right)}$$

p = saturated vapor pressure; p_c_ = critical pressure (22.064 MPa); T_c_ = Critical temperature (647.096 K); a_1_ = −7.859; a_2_ = 1.844; a_3_ = −11.786; a_4_ = 22.680, a_5_ = −15.961, a_6_ = 1.801 and τ = 1-(T + 273.15)/T_c_; e and e_s_ are actual and saturated vapour pressure respectively.

**Fig. 4:**
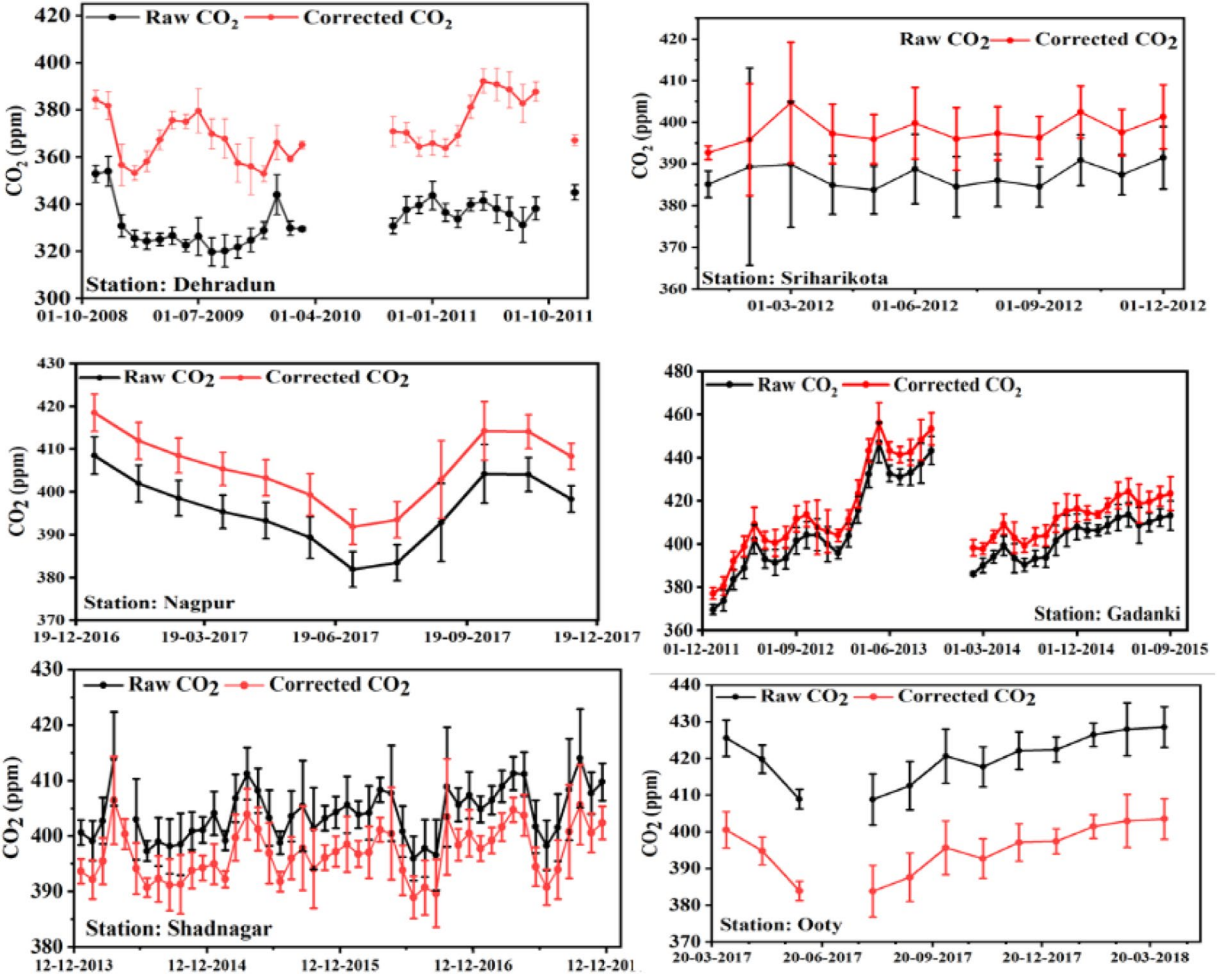
Water vapor corrected monthly mean of atmospheric CO_2_ variation over the study locations.

Figure 4 displays the monthly water vapour corrected atmospheric CO_2_ concentration compared to the raw atmospheric CO_2_ concentration (wet CO_2_) over the observational sites for the corresponding periods. The nearly uniform difference in atmospheric CO_2_ content between wet and dry is noticed at the stations. The relative bias between the dry and wet atmospheric CO_2_ concentrations are −11.27%, −2.80%, −2.52%, −2.24%, 1.78% and 5.95% over the Dehradun, SHAR, Nagpur, Gadanki, Shadnagar and Ooty respectively.

## Data Records

**Fig. 5:**
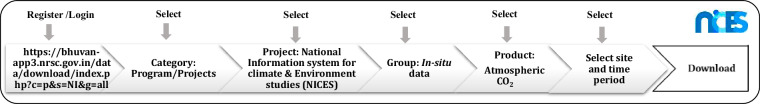
Flow chart for the CO_2_ data access from the NRSC/Bhuvan Portal of NICES.

We have made an effort to maintain synchronized atmospheric CO_2_ observations across the country. Data gaps over the study sites are due to technical snags in the instrument. Atmospheric CO_2_ data records are located at figshare online repository^28^ and the National Information system for Climate and Environment Studies (NICES) web page under the ISRO’s Bhuvan Geo-portal platform (https://bhuvan-app3.nrsc.gov.in/data/download/index.php). Data can be downloadable to the login users only. After login, the procedure to download the data has been given in Fig. 5.

Atmospheric CO_2_ data is formatted in a single Microsoft Excel (.xlsx) file. The first sheet is labelled as “daily_Atmospheric_CO2_ppm”, in which the first column represents the date in DDMMYYYY format. From the second to eighth columns are CO_2_ measurement locations, namely Dehradun, Gadanki, SHAR, Ponmudi, Shadnagar, Nagpur, and Ooty, respectively. The second sheet of the file contains the daily raw CO_2_ labelled as “Raw_daily_CO2_ppm”. Third sheet contains hourly corrected atmospheric CO_2_. Gaps in the meteorological data are obtained from a fifth generation European Centre for Medium-Range Weather Forecasts reanalysis (ECMWF-ERA5) climate data, an open access platform (https://cds.climate.copernicus.eu/cdsapp#!/dataset/reanalysis-era5-single-levels). Fourth sheet tagged as “Meta_data_Info” depicts the information of the data records, such as station names with their geographical locations (Latitude and Longitude) and the respective data period. The fifth sheet, i.e., inter-sensor_calibration, contains the simultaneous data recorded by the UGGA and GMP-343 CO_2_ sensors for inter-sensor calibration along with their deviations. The data in the fifth sheet has three columns; the first column is measurement time in HHMM format, the second column is CO_2_ in ppm measured by the UGGA, the third column is CO_2_ in ppm recorded by the GMP-343 sensor and fourth column is deviation between UGGA and GMP-343 measurements. Missing values in the xlsx file are indicated by the −999.

GMP-343 recorded CO_2_ data are formatted in a CSV file. Table 3 shows the study site’s positions, mean sea level (altitude), and data availability. At study sites, GMP-343 CO_2_ sensors are installed during different periods.

**Table 3:** GMP-343 CO_2_ sensor locations and data availability.

Station name	Latitude (N)	Longitude (E)	Altitude (m)	Data period
IIST, Ponmudi	8°45′	77°06′	1100.0	June 2014-May 2015
IISWC, Ooty	11°24′	76°40′	2240.0	May 2017- July 2021
SHAR, Sriharikota	13°43′	80°13′	9.0	January 2012-December 2012
NARL, Gadanki	13°27′	79°10′	375.0	January 2014-December 2014
NRSC, Shadnagar (GGA)	17°01′	78°11′	650.0	January 2014-December 2017
RRSC, Nagpur	21°09′	79°01′	310.0	January 2017-December 2017
IIRS, Dehradun	30°20′	78°02′	690.0	November 2008-December 2011

## Technical Validation

**Fig. 6:**
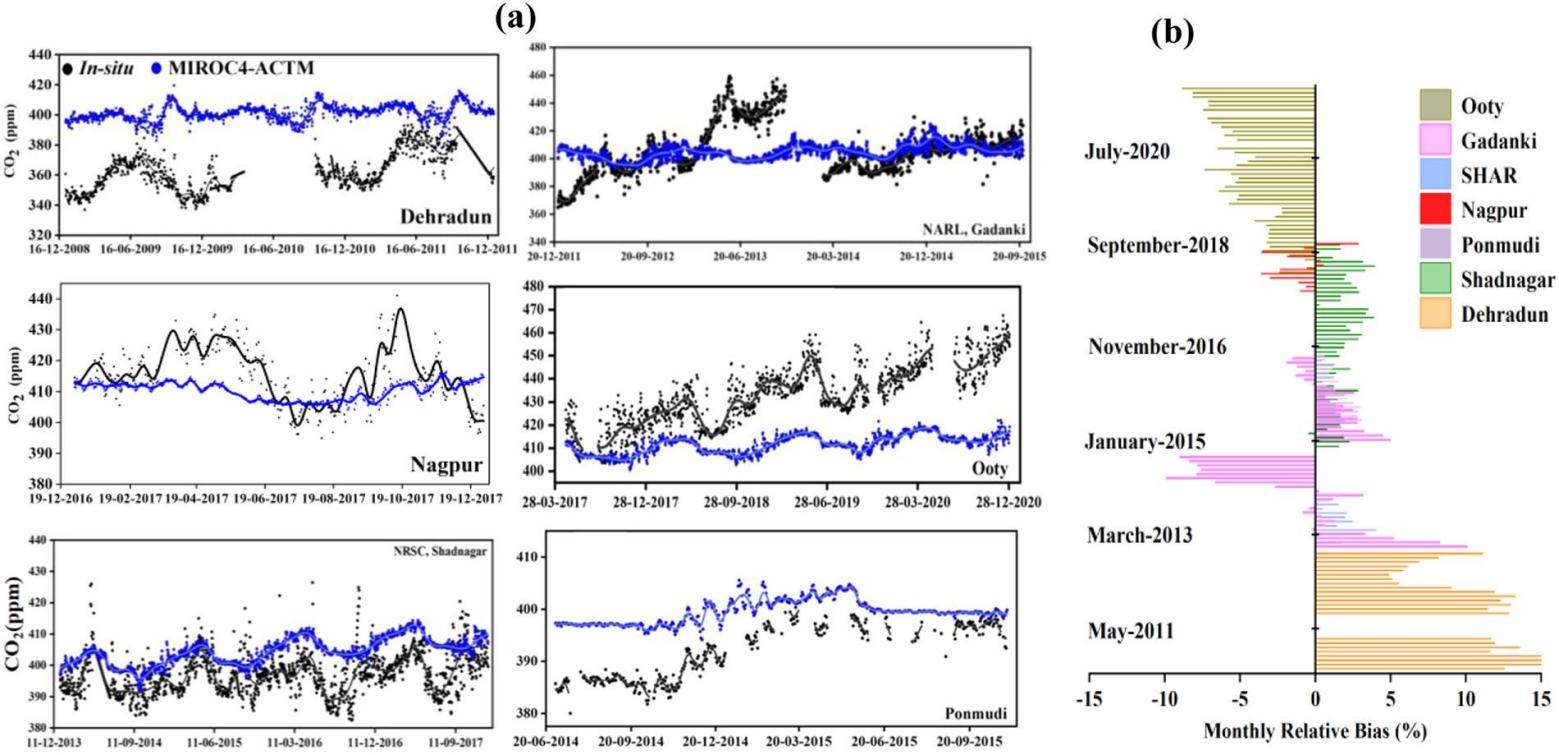
(**a**) Comparison of *in-situ* (dry) CO_2_ against the MIROC4 ACTM simulated surface level CO_2_ (**b**) Relative bias between monthly averaged *in-situ* CO_2_ against the MIROC4 ACTM simulation.

To maintain the quality measurements of continuous atmospheric CO_2_ observations, the GMP-343 CO_2_ sensors and the high precision greenhouse gas analyzer were periodically calibrated. An inter-sensor calibration was carried out between the GGA and GMP-343 CO_2_ sensors. An accuracy of the measurements from site to site are varied between 0.25% to 6.50%. The dry atmospheric CO_2_ is mainly controlled by the atmospheric pressure, temperature, and water vapor, hence atmospheric dilution correction was carried out. Due to the atmospheric dilution, observed a deviation of 3 ppm to 50 ppm at different locations. Further, the CO_2_ simulations from the Model for Interdisciplinary Research on Climate version 4^29^ (MIROC4); atmospheric general circulation model (AGCM)-based chemistry-transport model^30^ (referred to as MIROC4-ACTM;) are used to evaluate the *in-situ* observations against the model simulation for the Indian sites (Fig. 6). Observation from 50 sites across the globe are used for optimizing biospheric and oceanic fluxes. Detailed information about the simulations can be found in Patra *et al*.^31^.

The model-simulated CO_2_ and the baseline measurement over Nagpur match well; however, from March to April, there are noticeable increases in atmospheric CO_2_ concentrations. Measurements of Gadanki between 2011 and 2013 occasionally found inconsistent values. However, measurements in 2014 showed a good correlation with the model’s output. In the month of June 2013, anomalous measurements of atmospheric CO_2_ concentrations were recorded due to the technical snag of the instrument The mean monthly bias between *in-situ* CO_2_ against the MIROC4−ACTM simulated CO_2_ indicated the largest bias for Dehradun compared to other stations and systematic bias for Shadnagar (Fig. 6b). Overall, the bias between the dry corrected CO_2_ and model simulated CO_2_ lies within ±10%. Results of the comparison indicates the potentiality of the *in-situ* CO_2_ for the use of atmospheric research.

## Data Availability

There is no specific custom code used to generate the data/figures presented in this work.
